# Strategy for Deployment of Integrated Healthy Aging Regions Based Upon an Evidence-Based Regional Ecosystem—The Styria Model

**DOI:** 10.3389/fmed.2020.510475

**Published:** 2020-09-29

**Authors:** Marcus Borrmann, Sonja Lindner, Kathrin Hofer-Fischanger, Robert Rehb, Katrin Pechstädt, Roswitha Wiedenhofer, Gabriele Schwarze, Eva-Maria Adamer-König, Robert Mischak, Karl P. Pfeiffer, Johann Harer, Katharina Weinzerl, Christian Hartmann, Bernhard Rupp, Regina E. Roller-Wirnsberger

**Affiliations:** ^1^Department of Health Studies, FH Joanneum University of Applied Sciences, Graz, Austria; ^2^Department of Internal Medicine, Medical University Graz, Graz, Austria; ^3^Human.technology Styria GmbH, Graz, Austria; ^4^Joanneum Research Forschungsgesellschaft mbH, Graz, Austria; ^5^Center for Public Health, Medical University Vienna, Vienna, Austria

**Keywords:** active and healthy aging, reference site, ecosystem, deployment, health services and products

## Abstract

In 2013, the European Commission founded the platform European Innovation Partnership on Active and Healthy Aging as a communication and innovation network in this domain. The goal of the current study was the development of an integrated regional ecosystem for active and healthy aging for the region of Styria via a step-by-step co-creation process. A mixed model approach was used to establish an ecosystem for active and healthy aging, which includes macro-, meso- and micro-level stakeholders in the province of Styria, Austria. Based on the results, eight recommendations for the deployment of a healthy aging region were developed. The visibility and accessibility of healthy aging products and services were evaluated as key factors for innovation in active and healthy aging in the region. Health professionals were identified as major drivers of innovation related to active and healthy aging in Styria. The study presented in this article assessed the capacities for healthy aging in the Styria region and identified the need to improve communication pathways between all levels of the public health system and market.

## Introduction

Longevity is one of the main achievements of modern societies. Currently, almost one quarter of the European population is 60 years and older (1). The European Union has launched numerous initiatives to adapt to changes in the social, medical and economic needs driven by this demographic shift. A special emphasis has been placed on prolonging “active and healthy life-years” across Europe (2).

In this context, in 2013 (3) the European Union (EU) launched the European Innovation Partnership on Active and Healthy Aging (EIP/AHA) with a major goal of encouraging a broad partner and stakeholder engagement. Several calls for individual partner and regional commitments have been launched. To date, the EU has awarded 77 European regions the status of “reference sites for active and healthy aging” (4), which are coordinated and deployed under the umbrella of the Reference Site Collaborative Network (RSCN) (5). Reference Sites (RS) are “ecosystems which comprise various players (including regional and/or local government authorities, cities, hospitals/care organizations, industry, SMEs and/or start-ups, research and innovation organizations including universities and civil society), that jointly implement a comprehensive, innovation-based approach to active and healthy aging, and can give evidence and concrete illustrations of the impact of such approaches on the ground” (5, 6).

However, to date no recommendations for healthy aging regions (HARs) have been issued that describe the structured and evidence-based development and process of establishing and sustaining regional ecosystems based upon the model provided by the RSCN. A pilot study presented in this publication addresses this issue and describes the co-creation process of an ecosystem for active and healthy aging, including an indicator system for further monitoring of the awarded reference site Styria in southeastern Austria, Europe. The region concerned has about 1.24 million inhabitants with 20.1% of the population aged over 65 years. The population density varies from 24 inhabitants/km^2^ in the rural northern area to 260 inhabitants/km^2^ in the urban central area. Gross value added is structured in 1.9% of agriculture and forestry (primary sector), 33.2% industry, energy and construction (secondary sector), and 64.9% services (tertiary sector). The Styrian research and development rate amounts to 5.14%, which is the highest quota of Austria (7). The project presented in this publication was funded by the Styrian government following the award of the region as HAR by the European Commission in 2013.

## Methods

**Figure 1:**
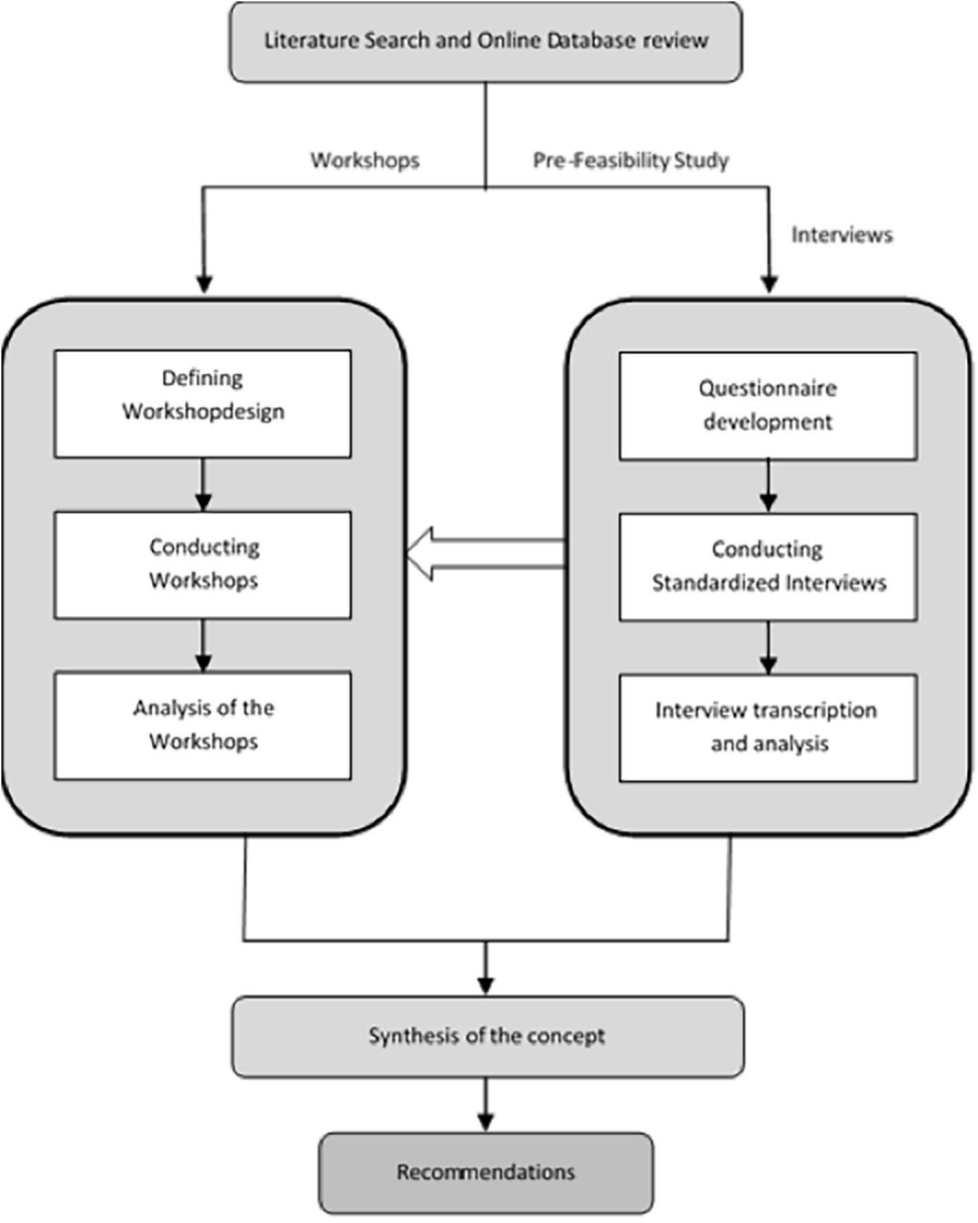
The method to develop an integrated ecosystem for active and healthy aging in the reference site Styria.

To achieve a comprehensive state of the art in active and healthy aging ecosystems, the authors of this publication selected a mixed model approach. The goal of this integrative procedure was to ensure a rigorous scientific research in the field of the development of an active and healthy aging ecosystem and to detect capacities, strengths and communication processes within micro-, meso- and macro-level of the ecosystem. First, evidence on ecosystem building was gathered from the international scientific literature, which was subsequently used to kick-off a regional co-creation process to establish an integrated ecosystem for active and healthy aging in the province of Styria. The entire process is explained in detail in the following Method Section and outlined in Figure 1.

As Figure 1 indicates, the process included three integrative steps: a comprehensive international literature review and a review of regional databases (applying identical key words); a qualitative research, including data collection via workshops and interviews; and a consensus-building process for the regional ecosystem, including recommendations for future capacity building.

### Comprehensive Literature Review of Pre-existing Data on Development of Ecosystems for Active and Healthy Aging Regions

A comprehensive literature review of the databases Medline (via Pubmed), Cochrane Database of Systematic Reviews and ScienceDirect was conducted using the search terms “healthy aging,” “aging-related diseases,” “frailty,” “frailty prevention,” and “reference site” (Figure 1). Furthermore, a hands-on search of grey literature was performed using the same search terms. Only articles published in English and German languages were considered for further evaluation, regardless of the year of publication. In addition, publications addressing sustainable outcome parameters and key indicators for monitoring a healthy aging reference site were included in the analysis.

All relevant information from the literature search was analyzed using the STROBE-Statement (STrengthening the Reporting of OBservational studies in Epidemiology) (8) and assessed for relevance according to the search terms described. This initial methodological step was conducted within a timeframe of 2 months.

### Comprehensive Online-Review of Active and Healthy Aging Activities on Regional Level Addressing the Region of Styria, Austria

National and regional databases of ministries, chambers and cluster organizations were screened to identify stakeholders, companies, and organizations using the search terms in English and German language in accordance to those used in the international literature review. A pre-feasibility study aimed at clarifying the structural complexity of the regional ecosystem and subsequently explaining possible relations and communication pathways for a regional stakeholder analysis. It was performed using an online tool to create a power/interest matrix (9). Individual and organizational strengths and activities addressing active and healthy aging were analyzed by rating the organizations in terms of their influence and importance within the region on a scale from 1 to 10 and their commitment to active and healthy aging on a scale from −5 to 5 (10). Answers from five regional stakeholders participating in this pre-feasibility work were weighted and transferred into one file to provide a first overview of stakeholders within the region.

Based upon the results of pre-feasibility study, an interview questionnaire was developed for face to face interviews with local experts in the field of active and healthy aging. In this interview template the following discriminators were applied according to the various levels of integrative neighborhood (11) and regional stakeholders: (1) readiness of political stakeholders and organizational structures at meso- and macro-levels to deliver services and products supporting active and healthy aging of Styrian citizens; (2) potential for innovation within the entire innovation cycle (12); (3) scalability at the international level; (4) evidence for triple win; and (5) proof of concept for deployment and innovation. Altogether, this step required time resources of 2 months.

### Selection of Experts

National and regional databases of ministries, chambers and cluster organizations were screened to identify relevant stakeholders within the content analysis described before. A list of regional stakeholders as potential interview partners and workshop participants was produced. Out of this list, the invited experts were selected using a random generator.

### Expert Interviews

The semi-structured interviews were conducted, transcribed and analyzed according to a standardized protocol. Information from the transcripts of the interviews was categorized and summarized in inductive steps. The experts received the questionnaire prior to the interview. All interview partners were informed about anonymity and data privacy and gave their consent to the interviews. Each interview was divided into four sections based on the results of the stakeholder analysis and the level of an integrative region (micro-, meso- or macro-level). Potential contributions, barriers and the role of industrial partners were discussed. One of the key aspects in this section was how the various levels of stakeholders should communicate and how the recipients of healthy aging services and products should be informed. Further, the participants were asked how and where they would start with regard to the implementation of healthy aging actions. Interviews were conducted by one investigator (MB) within a timeframe of 3 months.

### Expert Workshops

Five interdisciplinary standardized workshops with representatives of regional stakeholders were conducted over 3 months. Groups were formed in a way that ensured heterogeneous distribution of stakeholders within the working groups. All workshops focused on the strengths and capacities that had been discussed during the regional reviews (see Comprehensive literature review of pre-existing data on development of ecosystems for active and healthy aging regions and Comprehensive online-review of active and healthy aging activities on regional level addressing the region of Styria, Austria and Figure 1) using the method of participatory learning and a standardized approach (13). The goal was to obtain consensual results during the integrative group discussions, which were set up as interdisciplinary thinking corners and thematically aligned with the focus areas identified in the pre-feasibility study. Results for the working groups were summarized and transcribed according to the method of Philip Mayring (14).

### Data Analysis

All stakeholder interviews (as described in Expert interviews) were transcribed according to a standardized protocol and adjusted using dialect and filler words since the data were translated from German to English. Translation and summarization were accomplished by two researchers independently via a qualitative analysis approach proposed by Mayring (14). The categories of transcripts were analyzed, abstracted and summarized accordingly. To increase the intra-coder agreement, the inductive analysis was repeated by the same investigator at the end of the qualitative work, increasing the validity of the summaries.

The results were transferred to the workshops and the focus was narrowed using the discriminators that were applied in the first online phase of the consultation as part of the stakeholder analysis (see Comprehensive online-review of active and healthy aging activities on regional level addressing the region of Styria, Austria), i.e., readiness of political stakeholders and organizational structures on meso- and macro-levels to deliver services and products supporting active and healthy aging of Styrian citizens, potential for innovation, including the entire innovation cycle, scalability at the international level. Feedback from the participants was subsequently grouped and another inductive analysis was performed following the workshops to increase the validity of the summaries.

Based upon the methodology applied during the expert interviews and workshops, precise recommendations on how to strategically deploy the reference site in future political actions were developed. For this purpose, the main results of the literature research, expert interviews and focus group discussions were outlined and concrete recommendations were developed (Table 1). The emphasis was put on how well the framework for action for the reference site Styria matched the criteria launched by the reference site collaborative network (RSCN) (5, 6). This final conclusive methodological step was conducted over 4 months.

**Table 1:** Recommendation for action toward an integrated ecosystem for AHA in Styria.

**Recommendation**	**Mentioned in scientific literature**	**Mentioned in interviews with experts**	**Mentioned in workshops with experts**
1. Further expanding of the visibility of the references site on micro-, meso-, and macrolevel	X	X	X
2. Proof of effectiveness of AAL-solutions and assistive technologies	-	X	X
3. Development of an integrated healthy aging strategy	-	X	X
4. Demand oriented development of innovative products and services for healthy aging	X	X	X
5. Intensify the coordination tasks in the reference site	-	X	X
6. Development of relevant regionally available training offers for health professionals	-	X	X
7. Implementation of proposed measures by the experts to achieve the identified goals of the reference site (key performance indicators—KPIs)	-	X	X
8. Integration of healthy aging actions in health tourism and leisure activities	-	X	X

*Eight recommendations for action to foster a sustainable ecosystem for AHA in Styria. Only two of the recommendations have been discussed in scientific literature so far: visibility of the ecosystem for AHA in the region and support of product development for the market within the ecosystem. The remaining six recommendations elaborated reflect regional requirements*.

## Results

### Results of the Literature Review

Overall, 11 articles were identified based on the search terms used. After screening and analyzing the reports using the STROBE-statement, data from 6 relevant publications were extracted for further use in the analysis process during the literature review (8). The remaining publications (*n* = 5) were excluded from this analysis due to missing description of the methodology used in the report. Sustainable outcome parameters and key indicators detected for the search terms used (also see Comprehensive literature review of pre-existing data on development of ecosystems for active and healthy aging regions) were as follows: (1) quality of life (11, 15); (2) self-reported health (15, 16); and (3) frailty in the older population (17); (4) the need for enforced communication (17) between all stakeholder groups; and (5) the importance of considering a very specific and individual situation regarding health, social and economic aspects (18, 19).

### Results of Regional Online Analysis of Active and Healthy Aging Stakeholders

**Figure 2:**
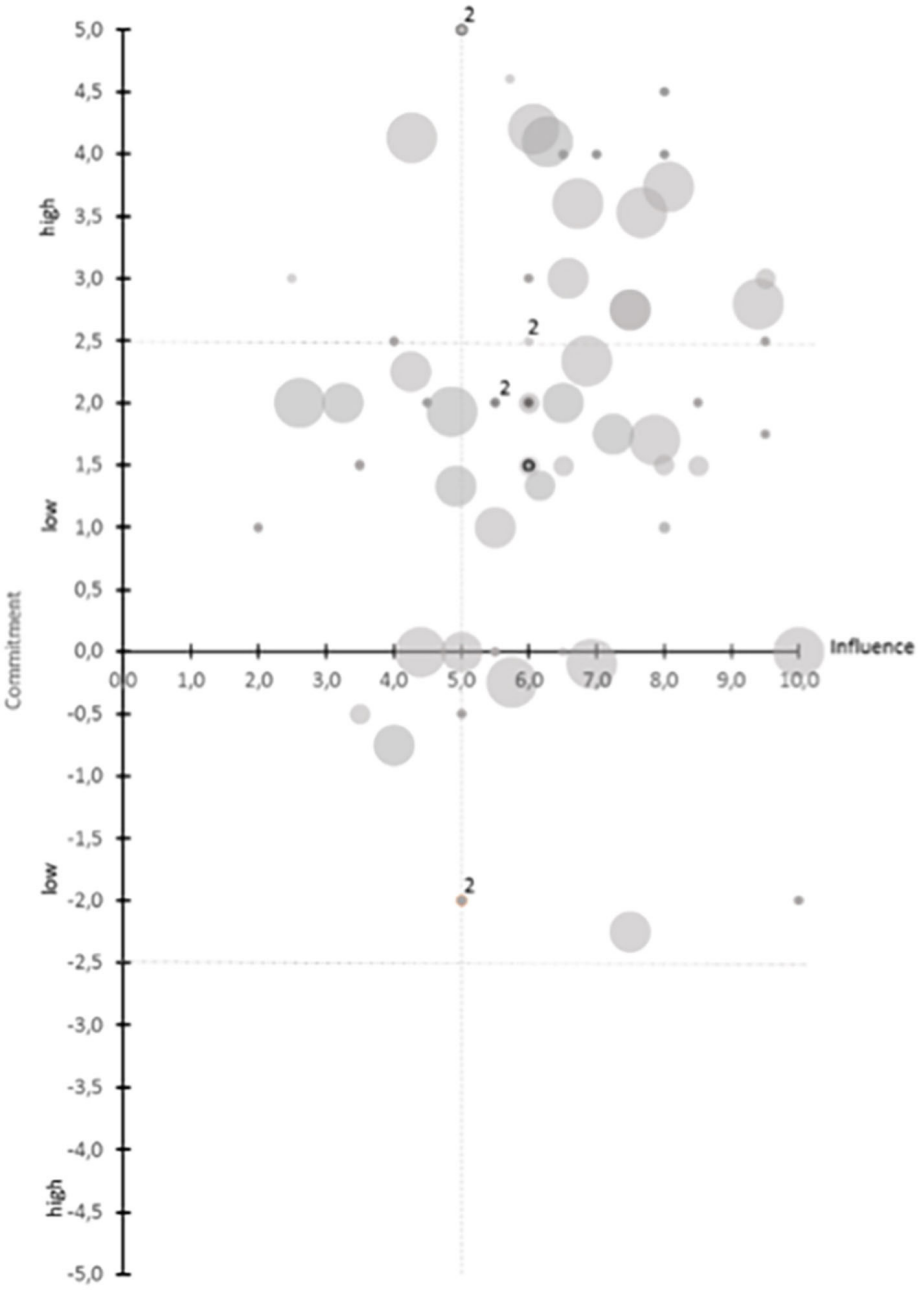
Results from the power/interest matrix (9) analysis condensed from feedbacks from 5 experts from the study core group of the current project. As may be seen from the figure, the method allowed analysis of individual commitments of different regional stakeholders and their effective influence on the development of an active and healthy aging region (HAR). This approach also allows to construct a taxonomy of regional stakeholders as primary “framework” of an ecosystem toward a HAR, not considering communication pathways at this point of the process management.

**Figure 3:**
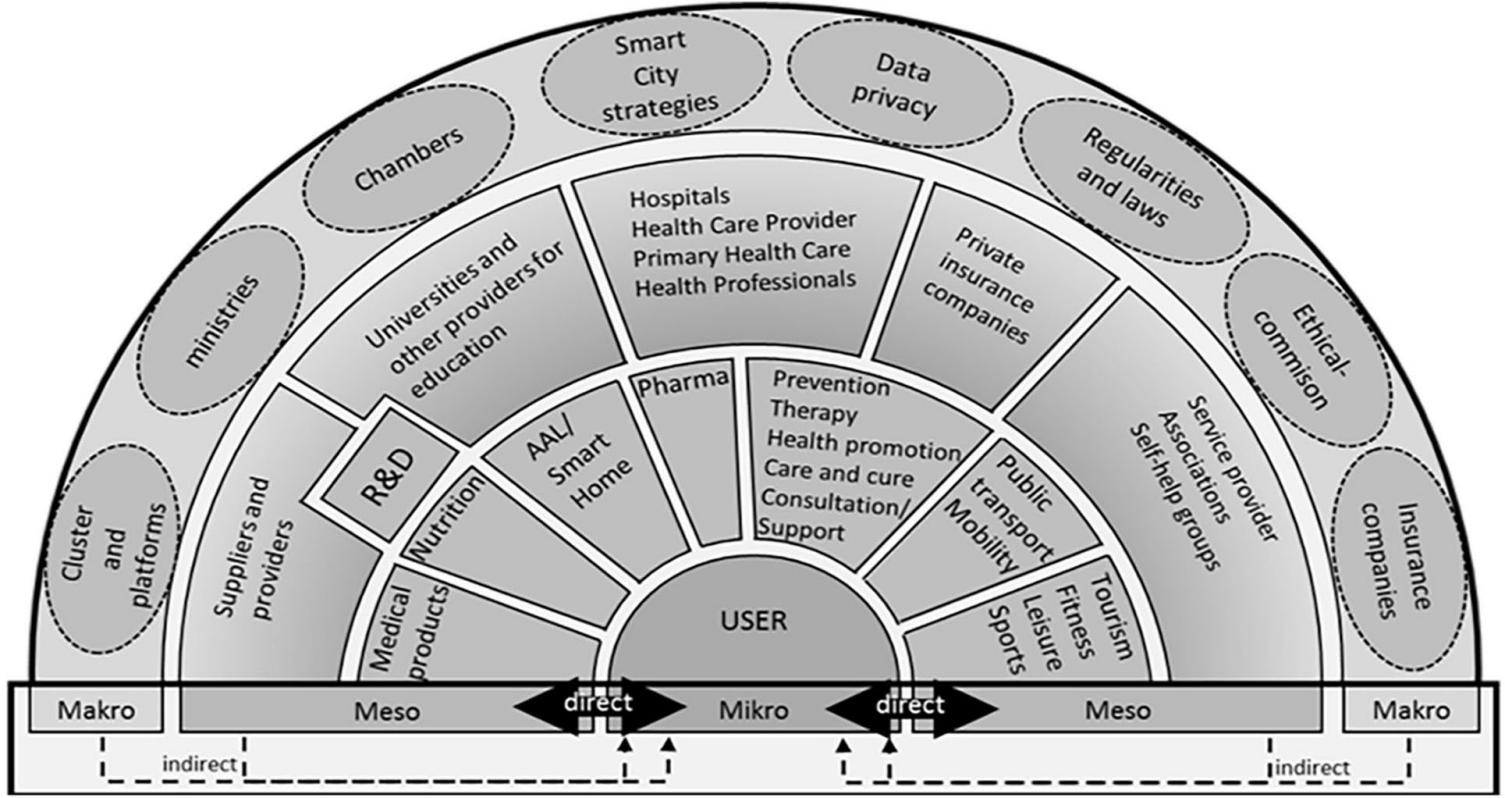
Humans, institutions and legal bodies and their position in the active and healthy aging ecosystem Styria according to results from the study presented in the current publication. In a mixed-model approach it was possible to condense information on regional structure and processes with evidence gathered during a literature search and feedback collected in face to face interviews and workshops with regional experts and stakeholders.

The initial data base search (online-review) revealed 63 institutes, companies and organizations which could be attributed to the topic of active and healthy aging and were subsequently structured and clustered at the various public health levels in the ecosystem (macro- and meso-levels). As shown in Figure 2, all stakeholders were categorized by the core team based on their role in the entire ecosystem and their proximity to the recipients of services and technologies. Figure 3 shows the direct communication pathway between health professionals and allied health professionals and their clients. In contrast, research organizations, such as universities and research companies that develop supportive assistance and technologies, have a more distant relation to end-users and citizens. To complete the list of relevant stakeholders, databases, and business directories of regional organizations for healthy aging services and products were analyzed, yielding additional 198 hits. The organizations identified include health and social services (*n* = 18), self-aid groups (*n* = 20), tourism and leisure providers (*n* = 3), local suppliers and mobility (*n* = 7), education and further qualification (*n* = 9), food supplement and pharma (*n* = 9), suppliers and services in the field of human technology (*n* = 65), education and research and development (*n* = 22), healthcare (*n* = 63), providers of related technologies (*n* = 26), representation of interests (*n* = 19).

### Results of Pre-feasibility Study of Stakeholders in the Future Ecosystem on Active and Healthy Aging

Research organizations, governmental organizations, associations and clusters are strongly committed to healthy aging and the Styrian EIP/AHA reference site. Data privacy organizations have a high influence in this field with respect to assisting technologies (7, 5/10). At the same time, data privacy aspects are considered a challenge to the development and implementation. In 14 ratings, all five experts provide their rating. In 35 of 63 ratings, one of the experts provided a rating for the organizations with activities in the field of healthy aging. Overall, within the Styrian EIP/AHA Reference Site the commitment to healthy aging activities is high (Figure 2).

### Results of Expert Interviews

Based upon results of the pre-feasibility study, 30 questions were included in the questionnaire for the expert interviews (data not shown).

Overall, 10 (5 male and 5 female) interview partners were invited and agreed to participate. One of the key messages conveyed during the interviews by the experts was a common understanding of healthy aging in Styria as one of the main strengths of the region. Furthermore, a high number of organizations active in this field represents a decisive regional strength. In addition, the experts described a requirement for improving visibility, transparency and communication related to the active and healthy aging reference site. There is a need to connect the stakeholders and to create collaborative initiatives in the region following a common strategy for active and healthy aging, focusing on structuring communication between the stakeholders (see Table 1 that summarizes all information from the three information sources). The key results shown in Table 1 represent prioritized spheres of activity, which were considered in at least two out of the three information sources (Table 1).

To support interaction leading to an integrated active and healthy aging ecosystem, the recipients of the services and technologies as well as the GPs, health professionals and formal and informal caregivers must be informed about the decisions and projects that directly concern them. Furthermore, including healthy aging aspects into the domains of housing, leisure time, mobility, and public transport was described to have high importance. Experts from research organizations would like to have evidence of the effectiveness and efficiency of assistive technologies for older people.

### Results of Workshops

Thirty three experts (16 male/17 female) agreed to participate in the workshops. Their professional background was as follows: health and social care professional, nursing professional, social services professional, a sociologist, medical doctors, managers, technicians, scientists, engineers, nurses, physiotherapists, an occupational therapist, a research manager, a health manager, public health experts, representatives of insurance companies, members of the Styrian chamber of commerce, the Styrian government, a business development professional, and a health economics expert.

The workshops narrowed the fields of strengths in the province of Styria. Following the workshops and based upon the clustering of capacities, the authors aligned the results along the ecosystem (Figure 3).

As shown in the Figure 3, in the ecosystem a direct connection and communication exist between the providing companies and organizations and the citizens concerned. Other organizations at the meso-level of the AHA regional ecosystem have an indirect connection with the target population. This structure indicated a “follower's role” of these institutions and services in the deployment of services and products for citizens. The same indirect link is observed for organizations at the macro-level, implying a lack of information transfer with regard to population needs and demands. For example, actions in the field of Research and Development (R&D), such as innovative solutions and products for AHA, were not visible to the recipients at the time of the project.

Based upon a structure provided by the RSCN, the experts in the focus groups clustered all activities detected in the ecosystem and identified major areas of strength: activities related to the prevention and early diagnosis of diseases, activities addressing environmental factors supporting active and healthy aging and, finally, care services and products.

### Recommendations for an Integrated Ecosystem for Active and Healthy Aging in Styria (AHA Styria)

Figure 3 depicts the structure and stakeholders involved in the ecosystem for active and healthy aging Styria (AHA Styria).

As shown in Figure 3, the ecosystem around citizens defined as “end-users” groups the stakeholders in the region according to their impact and support for Styrian citizens with regard to a healthy lifestyle and social integration. The meso-level includes the provision of preventive offers, mobility, nutrition, pharma and leisure offers, which are well developed in the region of Styria. Such direct citizen's empowerment reflects the regional capacities and culture and demonstrates that the proposed method is suitable for aligning the integrated AHA ecosystem with the local strengths.

The methodology used also allowed to analyze the communication pathways and rank the obtained information, resulting in precise recommendations for future developments in Styria.

Benchmarking the regional recommendations based upon the results of the interviews and workshops suggests that only two of the eight recommendations are reflected in the scientific literature: the visibility of AHA ecosystem in the region and the support of product development for the market within the ecosystem. The remaining six recommendations concern regional requirements and are as follows: a proof of effectiveness of AAL solutions and assistive technologies, the development of an integrative and politically binding integrated healthy aging strategy, coordination in the region, training offers for professionals involved in AHA, the development of regionally applicable key performance indicators (KPI) to measure the success of AHA actions and the integration of the topic of AHA into leisure activities and health tourism. Table 1 summarizes the action recommendations.

## Discussion and Strengths and Limitations of the Work

This work provides a methodology for developing integrated AHA ecosystems in the context of reference sites of EIP/AHA. To achieve this objective a mixed model approach (20) was used. By combining evidence from the international scientific literature with AHA regional capacities and stakeholder input, a structured co-creation process was established, which was led by academic institutions (21). It resulted in a set of recommendations (Table 1) on how to specifically address the needs of end-users or citizens with regard to AHA. The approach also allowed identifying regional strengths and capacities in the field of AHA. The inclusion of communication pathways made it possible to detect communication gaps and create a taxonomy within the regional AHA support systems and offers.

The first important finding of the work presented is the fact that evidence in literature on the topic of system development in the context of AHA reference sites is scarce. This is not surprising given the innovation factor of this construct launched for the first time by the EC in 2013. A lot of evidence is presented on micro-level for interventions and solutions supporting individual AHA. However, transfer of this knowledge and experience into daily practice of European citizens is still unstructured, often happens by fortune and systems are not systematically developed toward the aim of AHA. Despite the involvement of academia in AHA, the process of co-creation on system level is still not well established in the context of the EIP/AHA partnership. This fact makes the current project pioneering work which promotes integrated system development at the regional level.

The main objective of this work was to place stakeholders and citizens in the center and actively engage them in the process in order to generate ideas that reflect the status quo in our reference site (Figure 1). A web-based search of all regional stakeholders possibly involved in the system actions toward AHA offered an opportunity to learn more about them and to get to know them before inviting them to interviews and workshops. This approach to the development of markets (22) and community health services (23) has been proven effective in the literature. In their work on health promotion in systems, Naaldenberg et al. (24) describe health and well-being as the result of a series of complex processes during which an individual interacts with other people and the environment. The authors argue that health promotion and active and healthy aging are “not a straightforward technical process but a complicated and diffuse social process in which stakeholders have to work together and share information, ideas and decisions.” Our major contribution presented in this paper is the development of combined methodologies that take into account social interaction at many levels, introducing power/interest analysis as well as workshops and structured interviews. Using this bottom-up approach, it was possible to gain comprehensive insights into the regional system supporting health and AHA.

Our work also offers a broad view on the topic of AHA and allows further understanding of developments needed within health, care and social systems regionally. Aging is not related to health or illnesses exclusively. The ability to live independently and autonomously, making individual decisions, and planning leisure time plays a key role with regard to HARs. A principal goal of health promotion is to instigate changes at the individual level as well as in the social and physical environments, driving the entire system forward rather than fixing one component. Culture, sports and social integration are also aspects of an HAR that need to be considered. Health and social care, health services, self-health management, and senior tourism require ready models. Figure 3 shows the results of our project confirming this theory. Not only is the Styria region rich in natural resources for health and tourism, but local stakeholders feel the impact of these offers for a broad public access in the context of prevention and wellbeing to support healthy aging in the province of Styria. The proposed approach will help to stimulate the region's competitiveness in the international tourism sector and promote the development of sustainable, responsible and high-quality tourism. Ultimately, in line with the European Commission's perspectives and goals (25) it will consolidate the image and profile of Europe as home to sustainable and high-quality destinations.

Access to health services, care, cure and supporting technologies contributes efficiency and effectiveness to the process of healthy aging. The results of the interviews show that manufacturing companies could benefit in particular from the R&D strengths of universities, colleges, and research institutes in Styria. Interdisciplinary projects, diverse expertise in the field of developing technology and the healthcare professionals' knowledge of the target group can strongly complement each other. This result may also be viewed as system innovation, at least for the Styria region.

Based on the results of our work, a new structure of HAR can be established in the integrated neighborhood (11). New investigations of the EIP/AHA Reference Site illustrate that there is a need for a change in the communication between the meso- and micro-levels, which would promote knowledge transfer at all levels. New platforms and communication channels must be created in response to the demands of the people. Situated at the meso-level, developers of healthy aging services are not in contact with consumers of their products, e.g., AAL and Smart Home Solutions. Health Professionals, such as GPs, occupational therapists, physiotherapists, dieticians, care professionals and others, have a close connection with their clients. Detailed information about life circumstances, social relationships and socioeconomic status is required to provide recommendations to the clients. However, a close relationship and connection between health professionals and R&D organizations would allow incorporating the expertise and knowledge of the former into the development of new healthy aging services and products.

This implies the need for new communication pathways across the region to ensure citizen involvement in the topic of AHA. This is vital for high visibility of the topic of AHA in a region and ensures accessibility for many individuals. Structured exchange of information about innovation, information, projects and products (Figure 1) must become a key element for political action in the light of AHA. The major finding of our work was the lack of communication from top down and only little knowledge was available on individual needs of citizens for AHA in daily living. Making use of evidence-based methodologies like described in this paper will allow a citizen oriented and evidence driven political decision making for AHA.

Major strength of the work presented is the stringent co-creation process described. To the best of the authors' knowledge, this is the first description of the evidence-based development of an ecosystem for AHA in a region in Europe and it therefore reflects pioneer work. Readers get an overview on factors affecting AHA on all public health levels. Furthermore, authors were able to pinpoint the impact of communication pathways to facilitate accessibility of AHA offers within a region. The ecosystem presented in Figure 3 may further be used by readers to test their own regional capacities and start evolutionary processes.

Major limitation of the work presented is its regional perspective and its duration of more than 14 months of work. Despite the fact that authors aligned the work with recommendations released by RSCN, results presented here still reflect a very regional picture. This may limit the impact of results, however, the method presented may serve as best practice model for readers to implement AHA offers, products and processes in their environment as the methods used represent a comprehensive toolbox of methodologies present to get information locally and globally and may be useful at different opportunities.

## Data Availability Statement

The datasets generated for this study are available on request to the corresponding author.

## Ethics Statement

The Ethics Committee of the Medical University of Graz (Address: Auenbruggerplatz 2, 3. OG, 8036 Graz) waived the requirement for ethical approval for this study due to the method chosen without using or revealing any personal or patient data, in accordance with the national legislation and the institutional requirements.

## Author Contributions

MB, KH-F, RR, KP, RW, GS, E-MA-K, RM, KPP, JH, KW, SL, CH, BR, and RR-W contributed to project conception and conduct. MB wrote the first draft of the manuscript. RR-W, SL, and MB wrote sections and finalized the manuscript. All authors contributed to the article and approved the submitted version.

## Conflict of Interest

JH and KW are employed by Human.technology Styria GmbH and CH is employed by JOANNEUM RESEARCH Forschungsgesellschaft mbH. Human.technology Styria GmbH was involved in the study as coordinator of the reference site for active and healthy aging Styria and JOANNEUM RESEARCH Forschungsgesellschaft mbH as legal research entity. Both organizations and the affiliated authors acted in coordinating and researching roles and had no financial or commercial interest within the study. This study received funding from the Styrian government and publication fees are financed by FH JOANNEUM University of Applied Sciences and Human.technology Styria GmbH. The remaining authors declare that the research was conducted in the absence of any commercial or financial relationships that could be construed as a potential conflict of interest.
