# Supplementary material for: Strategy for Deployment of Integrated Healthy Aging Regions Based Upon an Evidence-Based Regional Ecosystem—The Styria Model
Source: Front Med (Lausanne). 2020 Sep 29;7:510475. doi: 10.3389/fmed.2020.510475 (PMC7550727; doi:10.3389/fmed.2020.510475)
Supplement: Supplementary file 1 [file Table_1.DOCX]

Supplementary Material

# Table 1: Workshop information

| **WS** | **Title** | **No of participants n =** | **Key information** |
| --- | --- | --- | --- |
| WS I | teaching, training and further education in a healthy ageing region | 10 | - HP as key-multiplicator - HP needs more fundamental knowledge about available AAL-Solutions to recommend the right solution in for the individual situation - promote health literacy - improve the empowerment of elder people |
| WS II | the perspective of users, acceptance and need for AAL and assistive systems in a healthy ageing context. | 6 | - increase the acceptance of AAL-Solutions in a community setting - lack of evidence for the cost-effectiveness of AAL-solutions - depends on the individual situation of the potential users - The user needs to be informed about the current regional offers - low threshold access to the demanded products and services |
| WS III | tourism and leisure time in the healthy ageing context | 6 | - high potential for digitalization in this field - the connection between offers in tourism, culture and leisure time should be combined with health-related aspects - new individual forms of health promotion and prevention of age-related disabilities |
| WS IV | Health Technologies, AAL, ICT in a Healthy Aging Region. | 15 | - cooperation between R&D organizations and industrial partners - prototyping and the evaluation can be a future task for R&D organizations - users and the multiplicators need to be involved in the whole process - the visibility of available solutions a quality brand for healthy ageing solutions and products in the region |
| WS V | politics and the strategy in Styria in a Healthy Aging Region | 9 | - focus on health promotion and prevention of age-related disabilities - interdisciplinary network - all players need to be committed in the sense of health in all policies |

**Supplementary Table 1.** Table 1 shows a detailed description of each workshop held with the number of participants and the key information derived.
